# Controversies in the Management of Borderline Resectable Proximal Pancreatic Adenocarcinoma with Vascular Involvement

**DOI:** 10.1155/2008/839503

**Published:** 2009-03-11

**Authors:** Olga N. Tucker, Mohamed Rela

**Affiliations:** The Department of Hepatopancreaticobiliary and Liver Transplant Surgery, King's College Hospital, Denmark Hill, London SE5 9RS, UK

## Abstract

Synchronous major vessel resection during pancreaticoduodenectomy (PD) for borderline resectable pancreatic adenocarcinoma remains controversial. In the 1970s, regional pancreatectomy advocated by Fortner was associated with unacceptably high morbidity and mortality rates, with no impact on long-term survival. With the establishment of a multidisciplinary approach, improvements in preoperative staging techniques, surgical expertise, and perioperative care reduced mortality rates and improved 5-year-survival rates are now achieved following resection in high-volume centres. Perioperative morbidity and mortality following PD with portal vein resection are comparable to standard PD, with reported 5-year-survival rates of up to 17%. Segmental resection and reconstruction of the common hepatic artery/proper hepatic artery (CHA/PHA) can be performed to achieve an R0 resection in selected patients with limited involvement of the CHA/PHA at the origin of the gastroduodenal artery (GDA). PD with concomitant major vessel resection for borderline resectable tumours should be performed when a margin-negative resection is anticipated at high-volume centres with expertise in complex pancreatic surgery. Where an incomplete (R1 or R2) resection is likely neoadjuvant treatment with systemic chemotherapy followed by chemoradiation as part of a clinical trial should be offered to all patients.

## 1. INTRODUCTION

Patients with pancreatic malignancy continue to have a dismal prognosis determined by the histological classification and extent of disease at the time of diagnosis [1]. The prognosis for histologically proven invasive pancreatic cancer is poor, with a 5-year-survival rate of 9.7% following resection, and overall median survival time of 8.6 months [1]. Invasive ductal adenocarcinoma is the most common epithelial tumour of the exocrine pancreas, of which tubular adenocarcinoma is the most common histological subtype [1]. The only potentially curative treatment for invasive ductal adenocarcinoma is surgical resection. However, only 10–20% of patients are candidates for resection as approximately 50% present with metastatic, and 35% with locally advanced surgically unresectable disease. Locally advanced, surgically unresectable proximal pancreatic tumours are defined as those that encase adjacent arteries including the coeliac axis, superior mesenteric artery (SMA) or both, or that occlude the portal vein (PV), superior mesenteric vein (SMV), or superior mesenteric portal vein (SMPV) confluence (Tables 1 and 2). This paper reviews the literature on the management of borderline resectable proximal pancreatic cancers with vascular involvement with reference to assessment of resectability, staging investigations, survival, pancreaticoduodenectomy (PD) with major arterial and venous resection and the role of neoadjuvant therapy.

## 2. DISCUSSION

### 2.1. Assessment of resectability

Determining resectability of the primary tumour is the most important goal in initial patient evaluation. High-quality computed tomography (CT) scanning can be used to classify pancreatic tumours into resectable (Stage I or II), locally advanced, surgically unresectable (Stage III), or metastatic disease (Stage IV). In recent years, with further advances in imaging techniques with multidetector CT optimized for pancreatic imaging, a new subset of tumours have emerged termed “borderline” or “marginally resectable” tumours blurring the distinction between resectable and locally advanced, surgically unresectable tumours [2, 3, 5]. There is currently no consensus in the reported literature on the definition or management of borderline resectable tumours. The National Comprehensive Cancer Network (NCCN) recently defined borderline resectable tumours of the pancreatic head and body as those with severe unilateral SMV/PV impingement, tumour abutment on SMA, gastroduodenal artery (GDA) encasement up to its origin from the hepatic artery (HA), tumours with limited inferior vena cava (IVC) involvement, short-segment SMV occlusion with proximal and distal vein patency, and colon or mesocolon invasion (Table 1) [2]. Tumours are defined as unresectable in the presence of proximal SMV occlusion up to the PV branches (Table 1) [2]. Neoadjuvant chemoradiotherapy is advocated by the NCCN for any tumour where an incomplete R1 or R2 resection is likely [2]. The M. D. Anderson criteria for borderline resectable tumours include those with encasement of a short segment of the HA amenable to resection and reconstruction without tumour extension to the coeliac axis, abutment of the SMA involving ≤180° of the arterial circumference, or short-segment occlusion of the SMV, PV, or SMPV confluence with normal SMV below and normal PV above the area of tumour involvement amenable to resection and reconstruction (Table 2) [3].

### 2.2. Staging investigations

Multidetector CT imaging is regarded as the optimal diagnostic and staging investigation [3, 6–11]. Freeny reported an accuracy rate of 95–97% for detection of pancreatic carcinomas, and 100% for predicting unresectability by helical CT [12]. In a series of 46 patients with a suspected pancreatic tumour, Catalano et al. demonstrated sensitivity, specificity, and accuracy of multidetector CT of 97%, 80%, and 96%, respectively, with correct prediction of unresectability with sensitivity of 96%, specificity of 86%, and accuracy of 93% [13]. However, the ability of helical CT to predict tumour resectability ranges from 57 to 88% [12, 14, 15]. Its major limitation is its low sensitivity in the detection of small-volume disease with nondetection of small-volume hepatic, serosal and/or peritoneal metastases. Despite advances in radiological imaging techniques 20–35% of patients thought to have resectable tumours have unsuspected metastases [15–18]. In many centres, laparoscopic staging is an integral component in the preoperative staging protocols of patients with radiological resectable disease to avoid unnecessary laparotomy. Biopsy of suspicious liver serosal or peritoneal metastases inaccessible or too small for interventional radiological techniques can be performed. Extra pancreatic extension of tumour with colic or mesocolic involvement can be determined. Improvements in technology and better patient selection have reduced the benefit of staging laparoscopy. However, it continues to consistently upstage patients with preoperative radiologically determined resectable disease with a benefit in determining resectability of 15–20% [19–25]. Peritoneal cytology enhances the sensitivity of staging laparoscopy upstaging an additional 8% of patients with positive cytology and advanced unresectable pancreatic cancer [26–28]. In addition, laparoscopic ultrasonography can be performed to improve the diagnostic accuracy of staging laparoscopy with evaluation of regional nodal disease, local vascular involvement, and to search for liver metastases [21, 25, 29–33].

Critics of staging laparoscopy believe inoperable disease secondary to local extension and vascular encasement can only be determined by laparotomy [34]. In addition, many argue that staging laparoscopy is of minimal benefit as patients with unresectable disease will subsequently require a bypass procedure for biliary or gastric outlet obstruction. Historically, reports of the development of obstructive jaundice in as many as 70% and gastric outlet obstruction in up to 25% of patients with unresectable pancreatic adenocarcinoma have supported the role of prophylactic bypass [35–37]. More recent reports suggest a lower incidence of biliary and gastric outlet obstruction [38]. Also, prophylactic palliative surgical bypass may be unnecessary. In a study by Espat et al., only 3% of 155 patients with laparoscopically staged unresectable histologically proven pancreatic cancer required a subsequent surgical bypass [39].

Other available investigative tools include mesenteric angiography, which has been abandoned in most centres in favour of multidetector CT, magnetic resonance techniques, transabdominal and endoscopic ultrasonography (EUS), and intraoperative intraportal endovascular ultrasonography (IPEUS) [3, 40, 41]. The combination of magnetic resonance cholangiopancreatography and magnetic resonance angiography has a reported accuracy of 89.7% in predicting resectability in patients with pancreatic head carcinoma [42, 43]. EUS has a role in the detection of pancreatic cancer, tissue confirmation of malignancy by EUS-directed fine needle aspiration (EUS-FNA), and determination of tumour resectability by assessment of locoregional tumour extension [44–50]. EUS has been advocated as the most sensitive method to assess small lesions <2 cm in diameter [47]. In a series by Brandwein et al., the finding of a focal hypoechoic mass without other abnormalities predicted malignancy in patients with ductal adenocarcinoma with an accuracy of 89%, specificity of only 22.2%, and sensitivity of 100% as all patients with a pancreatic mass were evaluated [46]. Findings at EUS predicted tumour resectability with an accuracy of 85%, specificity of 75%, and sensitivity of 100%, and EUS-FNA correctly detected malignancy with an accuracy of 65%, specificity of 100%, and sensitivity of 59.5% [46]. Sensitivity and specificity rates of EUS for confirming venous invasion range from 69% to 93% and 71% to 100%, respectively [51–53]. Sensitivities of 59.5% to 91% have been reported with EUS-FNA in the diagnosis of pancreatic malignancy [46, 54, 55]. To avoid the risk of tumour seeding along needle tracts EUS-FNA is preferable to percutaneous approaches. The presence of a cytologist at the time of the EUS with immediate evaluation of the EUS-FNA specimen increases the accuracy of the procedure. However, cytological interpretation of the tissue sample can be difficult due to the presence of reactive atypia or a benign appearance of pancreatic ductal cells in a well differentiated pancreatic adenocarcinoma.

Intraoperative intraportal endovascular ultrasonography (IPEUS) is employed in a limited number of centres to assess locoregional tumour extension, in particular PV invasion and invasion of the second portion of the pancreatic head nerve plexus [56, 57]. Invasion of the extrapancreatic nerve plexus is common in pancreatic head adenocarcinoma. In these cases, achievement of an R0 resection would necessitate complete dissection of the extrapancreatic nerve plexus and the nerve plexus around the SMA resulting in severe diarrhoea. Nakao et al. advocate the use of IPEUS to evaluate for extrapancreatic nerve plexus invasion. In the absence of invasion of the second portion of the pancreatic head nerve plexus preservation of the left semicircular nerve plexus around the SMA is recommended to prevent the occurrence of postoperative diarrhoea [57]. In the presence of extrapancreatic nerve plexus invasion particularly to the nerve plexus around the SMA radical resection is contraindicated [57].

### 2.3. Survival

The goal of surgery is to achieve an R0 resection. Margin resection status is an important prognostic factor, and a margin-positive resection predicts early recurrence and reduced survival [3, 40, 58]. Previous reports suggest that patients with a positive surgical margin following PD have a similar survival rate to patients with locally advanced, surgically unresectable disease treated nonoperatively with 5-fluorouracil-based chemotherapy and irradiation [10, 57]. However, in some series, statistically significantly longer overall survival rates have been reported after palliative PD for pancreatic head, neck, and uncinate process adenocarcinoma than palliative surgical bypass or nonoperative candidates when performed in high-volume centres with minimal morbidity and mortality, and combined with adjuvant chemoradiotherapy [59, 60].

Historically, major vessel involvement has been a contraindication to resection in patients with pancreatic adenocarcinoma. In 1973, Fortner described a surgical approach of regional pancreatectomy involving en bloc resection of peripancreatic soft tissue, regional lymph nodes with resection of the PV (Type I), or resection and reconstruction of a major artery (Type II) [61]. Although these extended resections achieved improved resectability rates, associated high morbidity (67%) and mortality (23%) with low survival rates (3-year-survival rate 3%) discouraged generalized adoption of major vessel resection and reconstruction. However, since the 1970s there have been major advances in radiological and surgical techniques resulting in improved preoperative staging, better patient selection, and reduced surgical morbidity and mortality [3, 62]. Perioperative mortality rates of less than 4% following PD are now achieved in high-volume centres [1, 40, 58]. In a consecutive series of 650 PD procedures performed between 1990 and 1996 at The Johns Hopkins Medical Institute, the mortality rate was 1.4%, with a reoperation rate for complications of 4%, and a mean hospital length of stay of 13 days [63]. No death was observed in the last 190 consecutive patients who underwent PD [63].

### 2.4. Pancreaticoduodenectomy with major arterial resection

Tumour encasement or abutment >180° of the arterial circumference of the SMA, or encasement of the coeliac or HA remain contraindications to resection (Tables 1 and 2) [2, 3]. Tumour encasement of the SMA or coeliac artery usually predicts extensive involvement of the mesenteric neural plexus with an inability to achieve a negative retroperitoneal resection margin even with radical extended surgery [3, 57]. PD with a histologically proven positive retroperitoneal margin performed as part of a standard or extended resection is associated with a median survival of less than one year [10]. Major arterial resection and reconstruction has been associated with a high operative mortality and morbidity in some series with poor long-term outcome [ 40]. A tumour is deemed unresectable if the HA is encased with no technical option for reconstruction due to extension to the coeliac axis/splenic/left gastric junction or coeliac origin (Tables 1 and 2) [2, 3]. Borderline unresectable tumours with arterial involvement include those with tumour abutment of ≤180° of the circumference of the SMA, tumour encasement of the GDA up to its originat the HA, or short-segment encasement/abutment of the common hepatic artery(CHA) or proper hepatic artery (PHA)typically at the GDA origin. Segmental resection of the HA with reconstruction is usually possible by primary end-to-end anastomosis because of the redundancy of the artery, or by an interposition graft. This limited involvement of the CHA/PHA is typically due to tumour extension in a cephalad direction along the GDA. More subtle findings on multidetector CT can be helpful in determining tumour resectability. The ability to achieve an R0 or R1 surgical resection is more likely in the presence of periarterial stranding rather than dense tissue involving the artery [3].

### 2.5. Pancreaticoduodenectomy with major venous resection

There are two important questions to ask when considering PD with PV resection. Can PV resection be performed safely and does SMV/PV involvement affect long-term survival? These questions have been addressed in the published literature. In contrast to arterial resection, PD with PV resection can be performed safely with no increase in perioperative morbidity or mortality compared to standard PD [40, 57, 58, 64, 65]. Although in some studies PV resection has been associated with longer operative time, higher blood loss, greater transfusion requirements, and a longer length of hospital stay, reported operative morbidity and mortality is comparable to standard PD [40, 58, 64–68]. A recent series by Yekebas et al. demonstrated no statistical difference in operative time, intraoperative blood transfusion requirements, vascular complications, in hospital morbidity or mortality rates in 128 patients who underwent en bloc vascular resection compared to 449 undergoing standard resection [11]. This series of 585 consecutive patients included those with pancreatic, ampullary, and distal common bile duct cancers who underwent potentially curative resection over an 11-year-period [11]. Final histopathology confirmed pancreatic ductal adenocarcinoma in 482 of 585 patients (82%) of which 100 (21%) underwent vascular resection [11]. In a series by Bachellier et al., one of 31 patients died of mesenteric venous infarction 4 days following PD with SMV-PV resection resulting in a mortality rate of 3.2% [69, 70]. Of 21 patients who underwent PD with SMV-PV resection in the same series from 1994 no postoperative deaths were observed [69, 70]. In Van Geenen's et al., series of 250 consecutive PDs 34 (16%) underwent SMV-PV resection, of which 28 had a tangential wedge and 6 a segmental resection [69, 70]. The overall mortality for the series was 1.2%, with a 0% mortality after SMV-PV resection in the 34 patients [69, 70]. Although the numbers are small, these reports demonstrate the feasibility and safety of PD with SMV-PV resection when performed in centres with acceptable overall morbidity and mortality rates following standard PD. In a recently published series by Nakao et al., major vessel resection was performed in 201 of 289 (69%) patients who underwent curative resection for invasive ductal carcinoma of the pancreas from 1981 to 2005 [40]. This group performs combined resection of major vessels including the PV and visceral arteries if involved with catheter bypass of the PV, extrapancreatic nerve plexus excision, and extended lymphadenectomy including the paraaortic lymph nodes with retroperitoneal connective tissue clearance. PV or SMV resection was performed in 200 patients, combined PV and arterial resection in 14, and hepatic arterial resection without PV resection in one patient [40]. Operative mortality was 3.8% for the 289 patients who underwent resection, 1.1% for patients without vascular resection, 2.7% for patients with SMV/PV resection, and 35.7% for combined PV and arterial resection [40]. The combined PV and arterial resection group had a higher operative death rate, more advanced tumours, and a higher incidence of positive dissected peripancreatic tissue margins [40].

Involvement of the SMV, PV, or SMPV confluence are not associated with histological variables predicting a poor prognosis [64]. Involvement of the SMPV confluence is thought to be a function of tumour location and size rather than an indicator of aggressive tumour biology [1, 64]. 5-year-survival rates of 7.4% to 17% have been reported in patients following PD with PV resection [1, 11, 57, 65]. However, 5-year-survival rates after PD with PV resection are significantly better after an R0 resection than an R1 or R2 resection [1, 40]. A higher incidence of perineural invasion and a greater median tumour size has been reported in a small number of studies in patients requiring PV resection [64, 65]. However, these variables have no statistically significant influence on survival when analysed in univariate or multivariate analysis [11, 65]. Importantly, patients who require PV resection in the absence of tumour extension to the SMA or coeliac axis have similar survival to patients undergoing standard PD [10, 71]. Nakao et al. have reported improved cumulative 5-year-survival rates in patients with tumour-free margins after PD with PV resection even in the presence of tumour invasion of the SMV-PV with a negative arterial margin [40]. Complete PV encasement with occlusion and thrombosis remains a contraindication to resection as arterial involvement is likely. It is important to note that PV resection in published series has been performed for clinical and/or radiological suspicion of PV involvement. Pancreatic adenocarcinoma is known to induce an extensive desmoplastic stromal reaction in surrounding tissues. Therefore, it can be difficult to distinguish true PV invasion from tumour adherence to the PV by an inflammatory reaction both on preoperative imaging and intraoperatively. Importantly, the absence of histological PV invasion is reported in 18% to 50% of cases with intraoperatively suspected vascular infiltration [11, 58, 65, 66, 69, 71–77]. Recently published series report peritumoural inflammatory adherence to the vein wall in 23% of patients suspected of vascular invasion [11, 71].

While PV resection itself is not a negative prognostic indicator, histologically proven PV invasion is independently correlated with lower patient survival [11, 57, 68]. Patients who undergo PV resection without true invasion of the vein wall have better survival than those with histologically proven PV invasion [11, 57, 68]. In the presence of negative dissected peripancreatic tissue margins, 2-year-survival is reduced in patients with histologically proven positive PV wall invasion following extended PD [57]. The degree of PV wall invasion correlates with outcome with reduced survival as the depth of invasion increases [68]. In a report by Nakagohri et al., 60% of patients with invasion of the tunica intima of the PV had extrapancreatic nerve plexus involvement [78]. In a recent study, Riediger et al. reported 5-year-survival rates following PD with PV resection for pancreatic head cancer of only 11% in the presence of histologically proven malignant PV invasion compared to 51% for those without venous invasion [58]. However, this result should be interpreted with caution as it represents a subgroup analysis in a total of 14 and 12 patients, respectively [58]. Other groups report equivalent median survival of patients with histopathologically confirmed vascular invasion compared to those without vascular invasion [11, 66, 76, 77].

### 2.6. The role of neoadjuvant therapy

To maximize the potential for an R0 resection, Varadhachary et al. advocate neoadjuvant treatment with systemic chemotherapy followed by chemoradiation in patients with borderline resectable tumours defined by the extent of local tumour growth on multidetector CT [3]. Following a 6 to 8 month course of neoadjuvant treatment, patients with responding or stable disease undergo PD with vascular resection if required. Varadhachary et al. describe their institutional experience with the use of differing protocols of neoadjuvant systemic therapy followed by chemoradiation, and four representative cases are presented [3]. Recent reports suggesting improved response rates, with acceptable tolerability and short-term outcome with gemcitabine-based chemoradiation neoadjuvant treatment protocols are promising [3, 79–83]. The optimal regimen of preoperative treatment has not been defined to date, and is the subject of ongoing clinical research trials. Detailed analysis of data from the Eastern Cooperative Oncology Group (ECOG 1200) phase II prospective multicentre study is awaited [84]. This study was designed to determine the efficacy of 2 neodajuvant chemoradiotherapeutic regimes, and the effect on margin-resection status in patients with locally advanced, potentially resectable pancreatic adenocarcinoma. Patients were randomized to receive neoadjuvant gemcitabine and radiotherapy or gemcitabine, fluorouracil, and cisplatin followed by radiotherapy and fluorouracil, followed by surgery and adjuvant chemotherapy [84]. Locoregional adjuvant chemotherapy may be of benefit in patients with positive PV margins following extended PD with PV resection. In a pilot study of postoperative intra-arterial chemotherapy improved survival to 25.6 months in patients following PV resection with histologically proven PV invasion was demonstrated versus 9.4 months without chemotherapy through reduction in the occurrence of liver metastasis [85]. Although the results of this study need to be interpreted with caution due to its small size further trials in this area are warranted.

## 3. CONCLUSION

Major vessel involvement should not be considered a contraindication to resection of borderline resectable pancreatic adenocarcinoma when a margin-negative resection is anticipated, and the procedure is performed in a high-volume centre with acceptable morbidity and mortality rates. In our institution, all patients with suspected SMV/PV involvement in the absence of distant metastatic disease, where segmental resection and reconstruction are feasible, are considered candidates for PD with concomitant major vessel resection. Tumour encasement of the SMA, coeliac or HA remains contraindications to resection due to the inability to achieve a margin negative resection, and high operative mortality and morbidity. Patients with limited involvement of the CHA/PHA due to tumour extension in a cephalad direction along the GDA are considered candidates for PD with concomitant major vessel resection where segmental resection and reconstruction are feasible with minimal morbidity and mortality. Neoadjuvant treatment with systemic chemotherapy followed by chemoradiation as part of a clinical trial should be offered to all patients with borderline resectable tumours when an incomplete (R1 or R2) resection is anticipated.

## Figures and Tables

**Table 1:** National comprehensive cancer network practice guidelines in oncology for pancreatic adenocarcinoma-v.1.2008: criteria defining resectability status [2].

Resectable
*Head/body/tail tumour*
(i) No distant metastases
(ii) Clear fat plane around coeliac and SMA
(iii) Patent SMV/PV

Borderline resectable

*Head/body*
(i) Severe unilateral SMV/PV impingement
(ii) Tumour abutment on SMA
(iii) GDA encasement up to origin at HA
(iv) Tumours with limited involvement of the IVC
(v) SMV occlusion, if of a short segment, with open vein both proximally and distally (unresectable if occlusion of the proximal SMV up to the PV branches)
(vi) Colon or mesocolon invasion
*Tail*
(i) Adrenal, colon or mesocolon, kidney invasion

Unresectable

*Head *
(i) Distant metastases
(ii) SMA, coeliac artery encasement
(iii) SMV/PV occlusion
(iv) Aortic, IVC invasion or encasement
(vi) Invasion of SMV below transverse colon
*Body*
(i) Distant metastases
(ii) SMA, coeliac, HA encasement
(iii) SMV/PV occlusion
(iv) Aortic invasion
*Tail*
(i) Distant metastases
(ii) SMA, coeliac encasement
(iii) Rib, vertebral invasion
*Nodal status*
(i) Metastases to lymph nodes beyond the field of resection.

GDA = Gastroduodenal artery IVC = Inferior vena cava PV = Portal vein SMV = Superior mesenteric vein SMA = Superior mesenteric artery.

**Table 2:** M. D. Anderson criteria for defining resectability status of pancreatic cancer [3, 4].

Resectable
(i) No distant metastases
(ii) No extension to the SMA; normal fat plant between the tumour and SMA
(iii) No extension to the coeliac axis or HA
(iv) Patent SMV/PV

Borderline resectable

(i) Tumour abutment ≤180° (≤50%) of the circumference of the SMA
(ii) Short-segment encasement/abutment of the CHA (typically at the GDA origin)
(iii) Short-segment occlusion of the SMV/PV with suitable vessel above and below

Unresectable

(i) Encased SMA (>180°)
(ii) Encased HA with no technical option for reconstruction
(iii) Occluded SMV/PV wih no technical option for reconstruction

CHA = common hepatic artery GDA = Gastroduodenal artery HA = Hepatic artery PV = Portal vein SMV = Superior mesenteric vein SMA = Superior mesenteric artery.
